# Supplementary figures and images for: Pathogenesis of Respiratory Syncytial Virus Infection in BALB/c Mice Differs Between Intratracheal and Intranasal Inoculation
Source: Viruses. 2019 Jun 3;11(6):508. doi: 10.3390/v11060508 (PMC6631102; doi:10.3390/v11060508)

# Sup Fig 1: Gating strategy cells from bronchoalveolar lavage

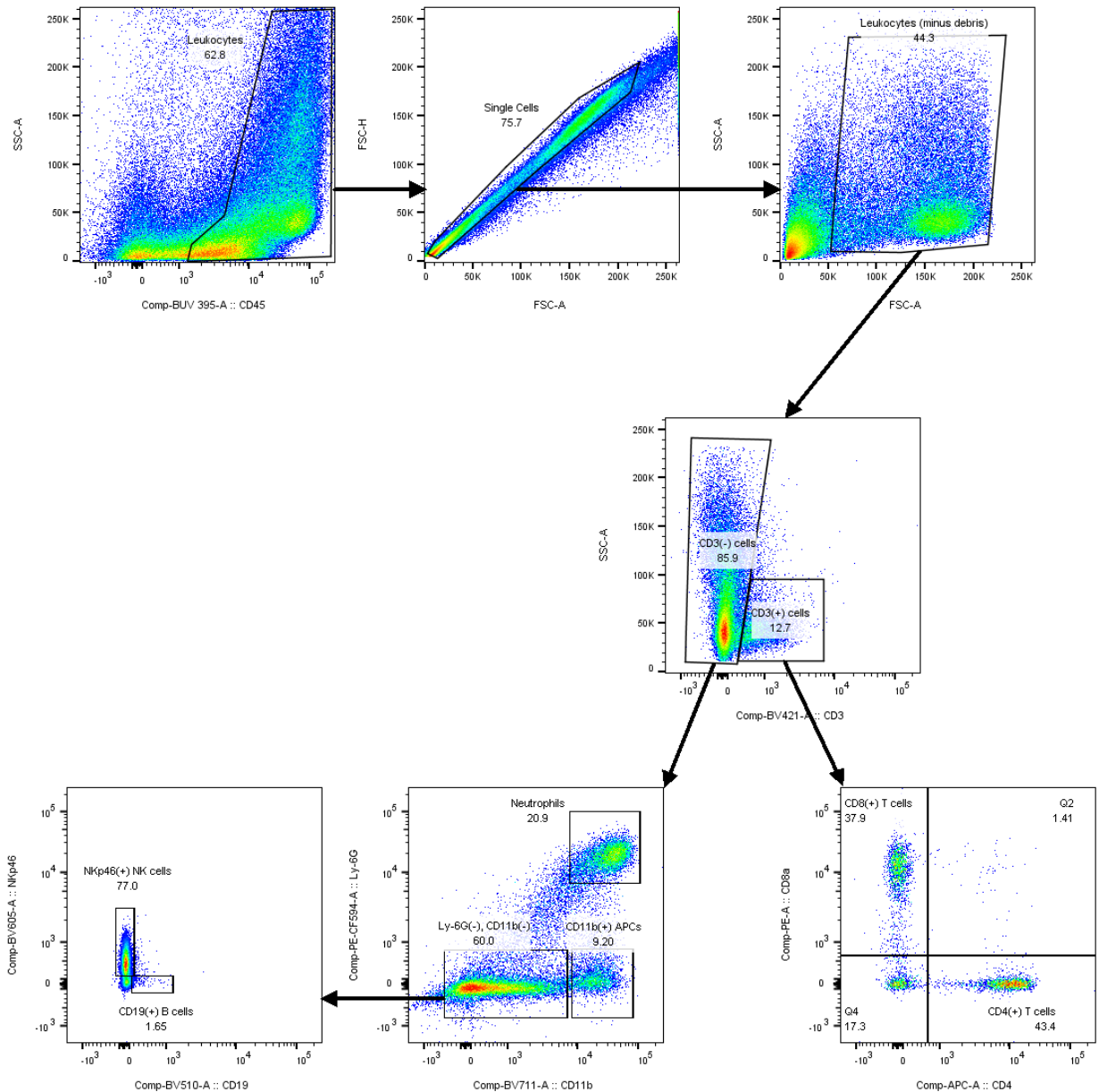

Supplement: Supplementary file 1 [file viruses-11-00508-s001.pdf]
